# Supplementary material for: Modeling glioblastoma heterogeneity as a dynamic network of cell states
Source: Mol Syst Biol. 2021 Sep 16;17(9):e10105. doi: 10.15252/msb.202010105 (PMC8444284; doi:10.15252/msb.202010105)
Supplement: Supplementary file 6 — Source Data for Figure 5 [file MSB-17-e10105-s004.zip › Figure5A_sourcedata/GSEA_3017/nosigdb_stateA.GseaPreranked.1622795026485/SUVA_AC_PMID_31327527.html]

Details for gene set SUVA\_AC\_PMID\_31327527[GSEA]

|  || Dataset | state53017 |
| Phenotype | NoPhenotypeAvailable |
| Upregulated in class | na\_neg |
| GeneSet | SUVA\_AC\_PMID\_31327527 |
| Enrichment Score (ES) | -0.69225466 |
| Normalized Enrichment Score (NES) | -2.9626253 |
| Nominal p-value | 0.0 |
| FDR q-value | 0.0 |
| FWER p-Value | 0.0 |
Table: GSEA Results Summary

  

Fig 1: Enrichment plot: SUVA\_AC\_PMID\_31327527      
 Profile of the Running ES Score & Positions of GeneSet Members on the Rank Ordered List

  

| PROBE | GENE SYMBOL | GENE\_TITLE | RANK IN GENE LIST | RANK METRIC SCORE | RUNNING ES | CORE ENRICHMENT || 1 | DBI |  |  | 17 | 0.683 | 0.0217 | No |
| 2 | S100A16 |  |  | 156 | 0.404 | -0.0982 | No |
| 3 | BCAN |  |  | 276 | 0.334 | -0.2024 | No |
| 4 | AGT |  |  | 587 | 0.252 | -0.5094 | No |
| 5 | S100B |  |  | 696 | -0.299 | -0.6043 | No |
| 6 | PON2 |  |  | 743 | -0.338 | -0.6325 | No |
| 7 | HEY1 |  |  | 751 | -0.344 | -0.6200 | No |
| 8 | SPARC |  |  | 782 | -0.369 | -0.6299 | No |
| 9 | MLC1 |  |  | 783 | -0.369 | -0.6086 | No |
| 10 | HOPX |  |  | 821 | -0.403 | -0.6238 | No |
| 11 | F3 |  |  | 888 | -0.526 | -0.6620 | Yes |
| 12 | GPM6B |  |  | 897 | -0.542 | -0.6391 | Yes |
| 13 | ATP1B2 |  |  | 904 | -0.558 | -0.6131 | Yes |
| 14 | MT3 |  |  | 905 | -0.559 | -0.5809 | Yes |
| 15 | RAB31 |  |  | 916 | -0.586 | -0.5576 | Yes |
| 16 | TTYH1 |  |  | 932 | -0.652 | -0.5356 | Yes |
| 17 | CST3 |  |  | 933 | -0.680 | -0.4965 | Yes |
| 18 | CLU |  |  | 938 | -0.685 | -0.4611 | Yes |
| 19 | FABP7 |  |  | 971 | -0.994 | -0.4371 | Yes |
| 20 | SLC1A3 |  |  | 973 | -1.097 | -0.3750 | Yes |
| 21 | HEPN1 |  |  | 975 | -1.110 | -0.3121 | Yes |
| 22 | PMP22 |  |  | 982 | -1.519 | -0.2309 | Yes |
| 23 | SPARCL1 |  |  | 984 | -1.810 | -0.1278 | Yes |
| 24 | GFAP |  |  | 987 | -2.255 | -0.0000 | Yes |
Table: GSEA details [plain text format]

  

Fig 2: SUVA\_AC\_PMID\_31327527: Random ES distribution      
 Gene set null distribution of ES for **SUVA\_AC\_PMID\_31327527**
